# Associations of central obesity and habitual food consumption with saliva microbiota and its enzymatic profiles – a pilot study in Finnish children

**DOI:** 10.3389/fmicb.2023.1323346

**Published:** 2024-01-08

**Authors:** Nitin Agrawal, Federico Fontana, Chiara Tarracchini, Sohvi Lommi, Marco Ventura, Christian Milani, Heli Viljakainen

**Affiliations:** ^1^Department of Public Health, Folkhälsan Research Center, Fin-HIT Research Group, Helsinki, Finland; ^2^Faculty of Medicine, University of Helsinki, Helsinki, Finland; ^3^Laboratory of Probiogenomics, Department of Chemistry, Life Sciences, and Environmental Sustainability, University of Parma, Parma, Italy; ^4^GenProbio srl, Parma, Italy; ^5^Microbiome Research Hub, University of Parma, Parma, Italy

**Keywords:** metagenomics, sugar, dairy, fruit and vegetables, bacterial species, oral microbiome

## Abstract

**Background:**

Variation in diversity and composition of saliva microbiota has been linked to weight status, but findings have been inconsistent. Focusing on clinically relevant conditions such as central obesity and using advanced sequencing techniques might fill in the gaps of knowledge.

**Aims:**

We investigated saliva microbiota with shallow metagenome sequencing in children with (*n* = 14) and without (*n* = 36) central obesity. Additionally, we examined the role of habitual food consumption on microbial enzymatic repertoire.

**Methods:**

Data comprised 50 children (50% male) with a mean age of 14.2 (SD 0.3) years, selected from the Finnish Health in Teens (Fin-HIT) cohort. Dietary scores for consumption frequency of sweet treats (STI), dairy products (DCI) and plants (PCI) were derived based on a self-administered food frequency questionnaire. Central obesity was defined based on waist–height ratio using the cut-off 0.5. Saliva samples were subjected to whole-metagenome shotgun sequencing, and taxonomic and functional profiling was achieved with METAnnotatorX2 bioinformatics platform.

**Results:**

Groups had an average 20 (95% CI 14–27) cm difference in waist circumference. We identified the lack of *Pseudomonas guguagenesis* and *Prevotella scopos, oulorum* and *oris* as putative biomarkers associated with central obesity and observed a total of 16 enzymatic reactions differing between the groups. DCI was associated with the highest number of enzyme profiles (122), followed by STI (60) and DCI (25) (Pearson correlation *p* < 0.05). Intriguingly, STI showed a high positive/negative correlation ratio (5.09), while DCI and PCI showed low ratios (0.54 and 0.33, respectively). Thus, the main driver of enzymatic reactions was STI, and the related pathways involved nitrate metabolism induced by *Haemophilus parainfluenzae* and *Veilonella dispar* among others.

**Conclusion:**

Clinically relevant differences in central obesity were only modestly reflected in the composition of saliva microbiota. Habitual consumption of sweet treats was a strong determinant of enzymatic reactions of saliva microbiota in children with and without central obesity. The clinical relevance of these findings warrants further studies.

## 1 Introduction

The prevalence of childhood overweight/obesity and obesity-related co-morbidities have been increasing at an alarming rate worldwide, posing a challenge to our well-being and healthcare system, even causing premature death (World Health Organization, 2022). The link between human microbiome and health conditions, obesity included, has been established but most of the microbiome research has focused on gut microbiota that likely contributes to human health by modifying nutrient intakes, preventing invading pathogens, and interacting with the immune system (Nishida et al., 2018; Lu et al., 2022). However, microbes reside in different niches of the human body: the oral cavity harboring the second most diverse microbiota after the gut (Deo and Deshmukh, 2019).

Saliva microbiota, in recent times, is gaining more attention as a first line of defense with similar contributions to human health as gut microbiota. Furthermore, as 1.5 liters of saliva is ingested daily, its role as an enhancer of gut colonization has also been proposed (Wade, 2021). Some studies have investigated saliva microbiota in overweight/obese children (Raju et al., 2019; Coker et al., 2022), but their results have been discordant with each other possibly due to differences in the study design, age range, and methodology. Moreover, none of these studies have considered central obesity that is mirrored by waist circumference or waist–height ratio (WHtR). WHtR is a better indicator of whole-body obesity than the traditionally used body mass index (McCarthy et al., 2005; Chrzanowska and Suder, 2010; Garnett et al., 2011), and intra-abdominal adipose tissue is known to function as an active organ producing hormones and cytokines which could potentially disrupt metabolic and inflammatory processes in the body (Mokha et al., 2010). In addition, the role of diet in oral microbiota has been scarcely studied in children. For example, frequent sugar consumption appears to play a role in the composition and functionality of the saliva microbiota (Lommi et al., 2022) but the associations between diet and enzymatic pathways of saliva microbial communities have so far not been examined.

While 16S rRNA gene profiling has been widely used to examine the human microbiome, it does not provide a reliable species-level identification. To overcome this shortcoming, metagenome sequencing methods are used that provide up to strain-level identification and thus, a deeper insight into the microbial population and its functional potential. In this study, we investigated central obesity and habitual food consumption as determinants of saliva microbiota composition. We compared composition and enzymatic properties of the saliva microbiota in participants with and without central obesity. Furthermore, we examined the enzymatic classes encoded by the salivary microbiome and the key taxa behind them. Saliva microbiota was subjected to shallow metagenome sequencing.

## 2 Materials and methods

### 2.1 Study population and data

For this study, we utilized questionnaire data, saliva samples, and anthropometric measurements available from the prospective Finnish Health in Teens cohort (Fin-HIT) which started in 2011 as a school-based cohort study, initially comprising of 11,407 Finnish children aged between 9 and 12 years living in large cities or their surrounding areas. The details of the Fin-HIT cohort are described elsewhere (Figueiredo et al., 2019). In 2015–2016, 54% of the children participated in the first follow-up by filling in an online health survey and providing a saliva sample. Here, we used data exclusively from the follow-up. The Coordinating Ethics Committee of the Hospital District of Helsinki and Uusimaa approved the study protocol (169/13/03/00/10), and written informed consent was obtained from all participants and their parents.

### 2.2 Anthropometrics

Children self-reported their height (cm), waist circumference (cm), and weight (kg) with instructions that the measurements should be done with the help from an adult. We have previously reported the validity of home-measured anthropometry among 113 children (Sarkkola et al., 2016). Waist–height ratio (WHtR) was calculated by dividing waist circumference (cm) with height (cm) after which children were categorized into those without central obesity (WHtR < 0.5) and those with central obesity (WHtR ≥ 0.5).

### 2.3 Food consumption and dietary scores

Three dietary scores were derived based on a self-administered food frequency questionnaire (FFQ) measuring consumption frequencies of 16 foods and drinks during the preceding month of data collection. The sweet treat index (STI) measures the weekly consumption of chocolate/sweets, ice cream, sweet pastries, biscuits/cookies, sugary juice drinks and sugary soft drinks (Lommi et al., 2020), the plant consumption index (PCI) that of vegetables, fruits and berries (Räisänen et al., 2022), and finally, the dairy consumption index (DCI) measured the weekly consumption of milk/buttermilk and ice cream. Our FFQ was adapted from the FFQ used in the WHO’s International Health Behaviour in School-Aged Children study, which was validated and retested among school-age children in Europe (Vereecken and Maes, 2003; Vereecken et al., 2008).

### 2.4 Sampling, sequencing and bioinformatic analyses of salivary datasets

Unstimulated saliva samples were collected using the Oragene^®^ DNA (OG-500) Self-Collection Kit (DNA Genotek Inc., Ottawa, Ontario, Canada). Saliva samples were mixed with a stabilizing reagent within the collection tube and stored at room temperature per the manufacturer’s instructions. After an intensive lysis and mechanical disruption protocol of microbial cells, genomic DNA was extracted using a CMG-1035 saliva kit and Chemagic MSM1 nucleic acid extraction robot (PerkinElmer) (Raju et al., 2018). Sampling was based on an equal number of normal-and overweight children with equal sex distribution. In total, 50 saliva samples were subjected to whole-metagenome shotgun (WMGS) sequencing on Illumina HiSeq 2500 system (Illumina Inc., San Diego, CA, USA) at the Institute of Molecular Medicine Finland (FIMM). The raw data in fastq format were submitted to quality filtering (quality score < 25) and for the removal of reads derived from the host. Subsequently, taxonomic profiling of the reads which passed filtering was achieved with the METAnnotatorX2 bioinformatics platform (Milani et al., 2021) using MEGABLAST employing the curated non-redundant sequence database of genomes retrieved from the National Center for Biotechnology Information (NCBI).

IBM SPSS v29 (IBM Corp., Armonk, NY, USA) and R studio (version 4.2.2)^1^ were used for comparing the Shannon index between the groups. Similarities between samples (beta-diversity) were assessed by the Bray–Curtis dissimilarity index based on species abundance. PCoA representation of beta-diversity was performed using OriginPro v2021 (OriginLab Corporation, Northampton, MA, USA), and analysed with permutational multivariate analysis of variance [PERMANOVA].

Microbial community functional analyses were performed using METAnnotatorX2, giving us enzymatic classification numbers by using the Metacyc database as a reference (Caspi et al., 2014). OriginPro v2021 was used to perform the statistical tests in each comparison of differentially abundant taxa and differentially encoded functional enzymes.

### 2.5 Statistical analyses

Background characteristics, dietary scores, and mean abundance of genera and species were compared between groups with and without central obesity with Mann–Whitney *U* test or Chi-Square using IBM SPSS v29. Results are presented as means with standard deviations (SD) or as counts (n) with percentages (%).

STI, PCI, and DCI correlation with enzyme repertoire was examined with Pearson correlation on R studio (version 4.2.2). Only significant correlations with correlation scores >0.2 or < −0.2 were retained to remove weak correlations from the results. Force-driven Network was used to represent correlation scores through Gephi (Version 0.9.6) software (Bastian et al., 2009) and ForceAtlas2 algorithm (Jacomy et al., 2014). Statistical significance was set at *p* < 0.05.

## 3 Results

### 3.1 Background characteristics

The participants’ background factors except for waist circumference did not differ between the groups (Table 1). Of the three dietary summary scores, only the dairy consumption index seemed to be non-significantly lower in children with central obesity than those without central obesity (*p* = 0.101).

**Table 1:** Background characteristics of groups without (no) and with (yes) central obesity with mean (SD), if not indicated otherwise.

	Central obesity				
	No^a^		Yes^b^		*p*-value^c^
n	36		14		
Sex, n (%)					1.000^d^
Female	18	(50.0%)	7	(50.0%)	
Male	18	(50.0%)	7	(50.0%)	
Age, y	14.2	(0.3)	14.3	(0.3)	0.778
Height, cm	167.1	(5.9)	168.4	(9.6)	0.509
Waist, cm	72.6	(5.5)	93.4	(10.7)	<0.001
Waist–height ratio (WHtR)	0.43	(0.03)	0.55	(0.05)	<0.001
Sweet treat index, times per week	7.6	(6.2)	6.1	(4.5)	0.404
Plant consumption index, times per week	15.1	(8.1)	13.2	(7.2)	0.523
Dairy consumption index, times per week	12.2	(4.7)	9.9	(5.7)	0.101

Waist–height ratio: calculated by dividing waist circumference (cm) with height (cm). ^a^No: without central obesity (WHtR < 0.5), ^b^Yes: with central obesity (WHtR ≥ 0.5). ^c^Mann-Whitney *U* test. SD; standard deviation. ^d^Chi-Square test.

### 3.2 Description of saliva microbiota in the entire sample

Whole-metagenome shotgun (WMGS) sequencing of the saliva microbiomes produced an average of 1.97 million ± 329,000 paired-end (2 × 150 bp) reads per sample. Following quality filtering and removal of reads that map on the *Homo sapiens* genome, an average of 285,567 microbial reads per sample, with a max of 1,427,437 reads were retained and subjected to downstream analyses (Supplementary Table 1).

**Figure 1:**
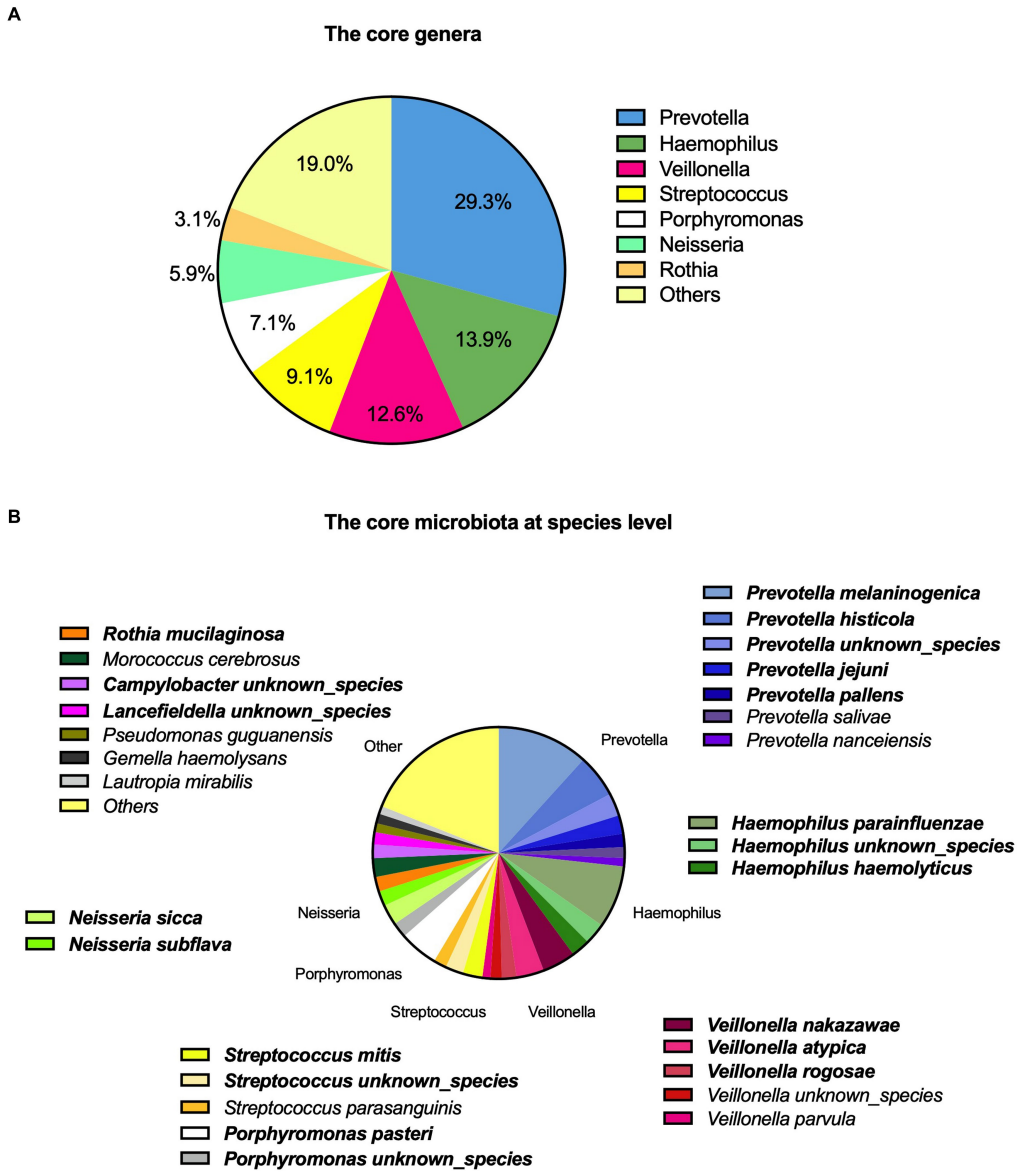
Description of saliva microbiota with **(A)** the core genera and **(B)** at a species level. The top 20 prevalent species are highlighted with bold.

In total, 43 genera were identified. On average, the participants had 15 genera present. The core microbiota consisted of seven genera based on the highest relative abundance across the samples as shown in Figure 1A, accounting for 81% of the total saliva microbiota.

We identified a total of 124 microbial species among the participants (Figure 1B). The top 20 species accounted for 69.5% of the total saliva microbiota, the top five being *Prevotella melaninogenica* (11.7%), *Haemophilus parainfluenzae* (8.0%), *Prevotella histicola* (5.5%), *Porphyromonas pasteri* (5.0%), and *Veillonella nakazawae* (4.3%). These accounted for 34.5% of the total. A complete description of the top 20 species is also shown in Supplementary Table 2.

### 3.3 Saliva microbiota composition according to central obesity

**Figure 2:**
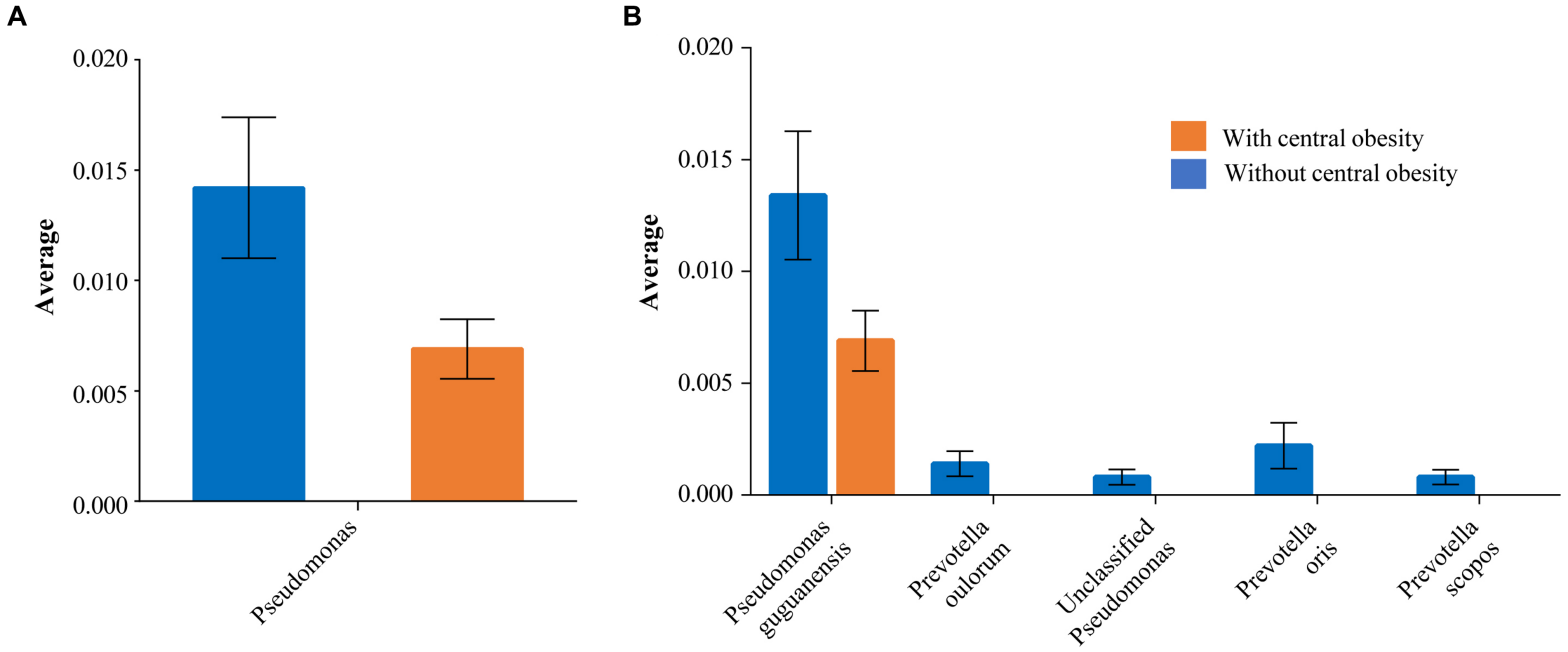
The taxa significantly differing between the groups with (orange) and without (blue) central obesity are shown with mean abundance for **(A)** genus and **(B)** species levels. The error bars represent SEM.

The Shannon index (SD) (alpha diversity) was similar between the groups: 2.99 (0.4) vs. 3.06 (0.3) (*p* = 0.53) with and without central obesity, respectively. The analysis of inter-sample variability (beta-diversity) did not reveal any compositional differences at genus level between the groups (*p* = 0.744). Notably, some taxa-specific differences were detected. *Pseudomonas* showed lower mean abundance in the group with central obesity than those without central obesity (0.7% vs. 1.4%, *p* = 0.041; Figure 2A and Supplementary Figure 1).

Similarly, a species-level investigation revealed that *Pseudomonas guguanensis* had a lower abundance among participants with central obesity compared with those without central obesity (0.7% vs. 1.3%, *p* = 0.046). Moreover, unclassified *Pseudomonas* and several *Prevotella* species, e.g., *scopos*, *oulorum* and *oris*, although present among those without central obesity, were completely absent in the group with central obesity (*p* < 0.05 for all; Figure 2B).

### 3.4 Enzymatic profiling – central obesity

To assess whether and how saliva bacterial community-derived metabolites differed between the groups, we analyzed the potential microbiota-encoded enzymatic profiles based on the MetaCyc (Caspi et al., 2014) database and the bioinformatic suite METAnnotatorX2 (Milani et al., 2021).

**Figure 3:**
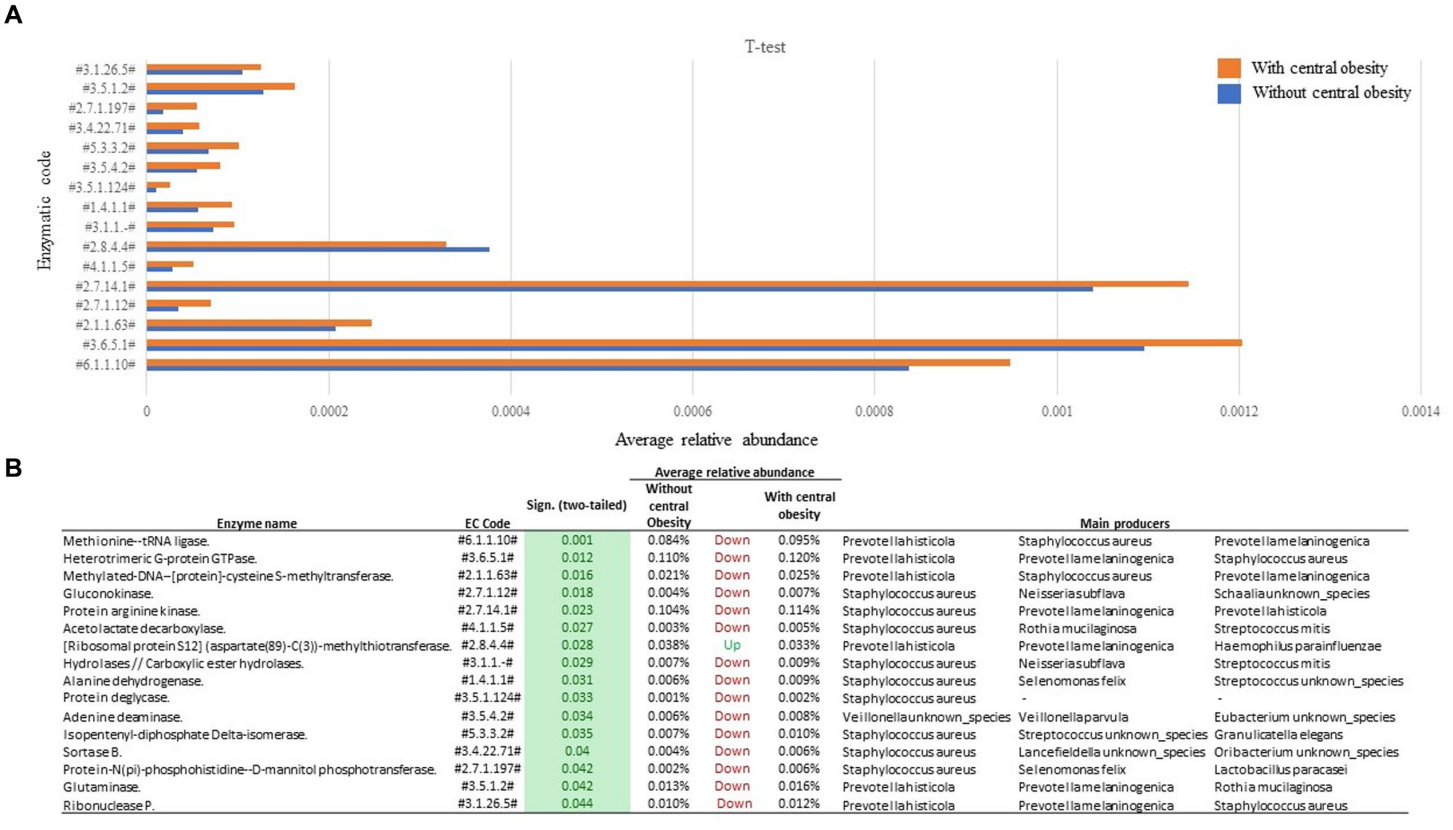
Functional analysis of saliva samples from participants with (orange, *n* = 14) and without central obesity (blue, *n* = 36) **(A)** revealed several differences in microbiota-encoded enzymatic potential profiles. **(B)** Table represents main producers of each enzymatic class.

A total of 16 enzymatic classes differed between the groups. Compared to the group without central obesity, group with central obesity showed enrichment in 15 enzymatic classes (Figure 3), while one enzymatic class was diminished. Through functional back-tracing of taxonomic information, we linked these classes to the microbial species that encode these enzymes. Overall, the analysis revealed that *Staphylococcus aureus*, *Prevotella histicola*, and *Prevotella melaninogenica* were the major taxa with the highest genetic potential for these enzyme-coding genes (Figure 3).

### 3.5 DCI, PCI and STI associate with enzymatic profiles

**Figure 4:**
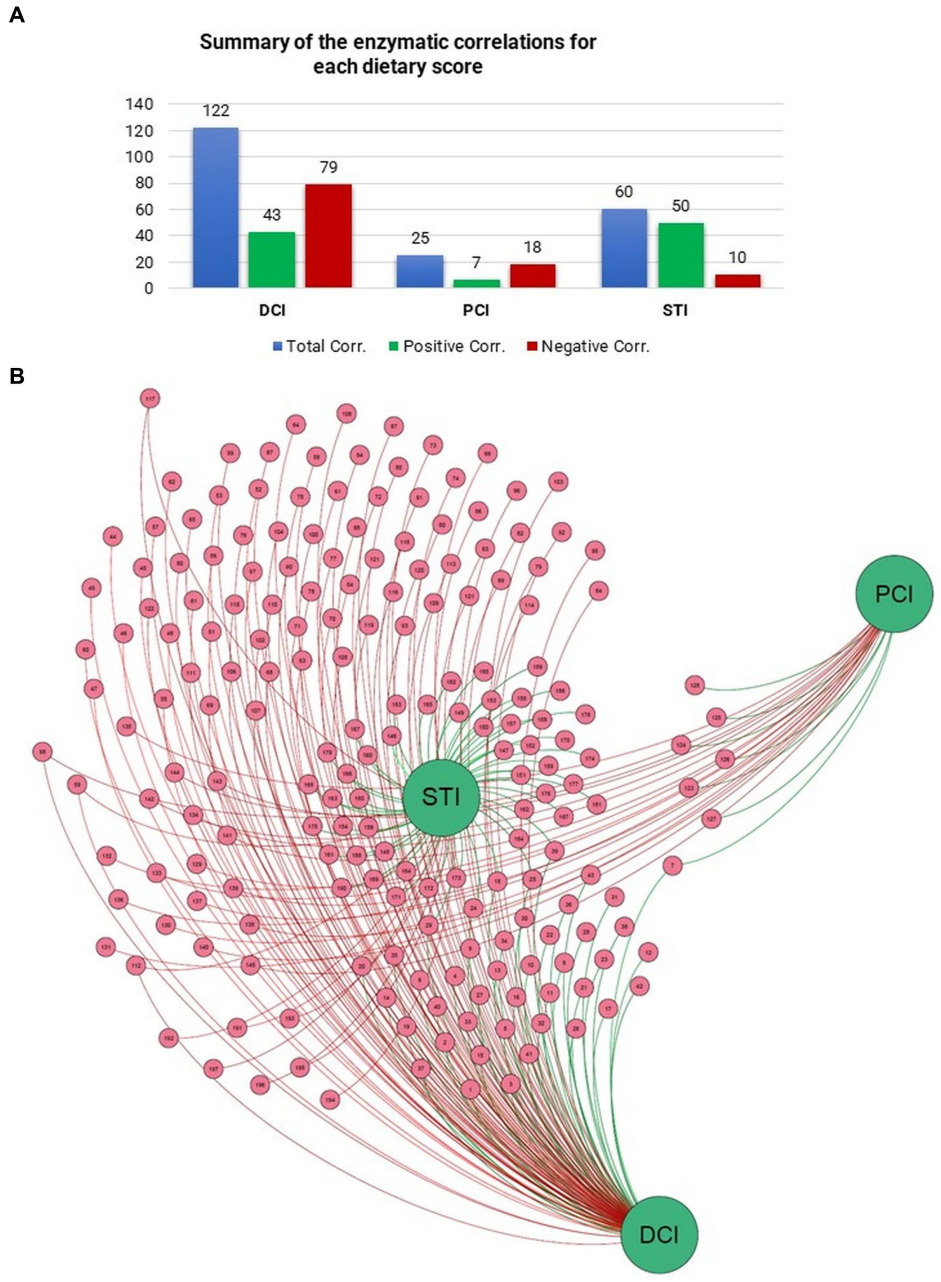
Number of Pearson correlations between **(A)** dietary scores and enzymes are presented in the table. **(B)** Force-driven network represents the entity of the correlation scores between dietary scores and enzymes through Gephi (Version 0.9.6) software and ForceAtlas2 algorithm.

To look for additional drivers for the enzymatic profiles, we examined correlations of DCI, PCI, and STI with relative EC numbers. DCI correlated with 122 enzymes, STI with 60, and PCI with 25 (*p* < 0.05). STI had a correlation ratio (positive/negative) of 5.09, DCI that of 0.54, and PCI that of 0.33 (Figure 4A). Figure 4B also shows the correlations of DCI, PCI, and STI with enzymes in the network representation. Thus, STI, with the highest correlation ratio and the most positive correlations with several enzymes, held a central position in the network.

**Figure 5:**
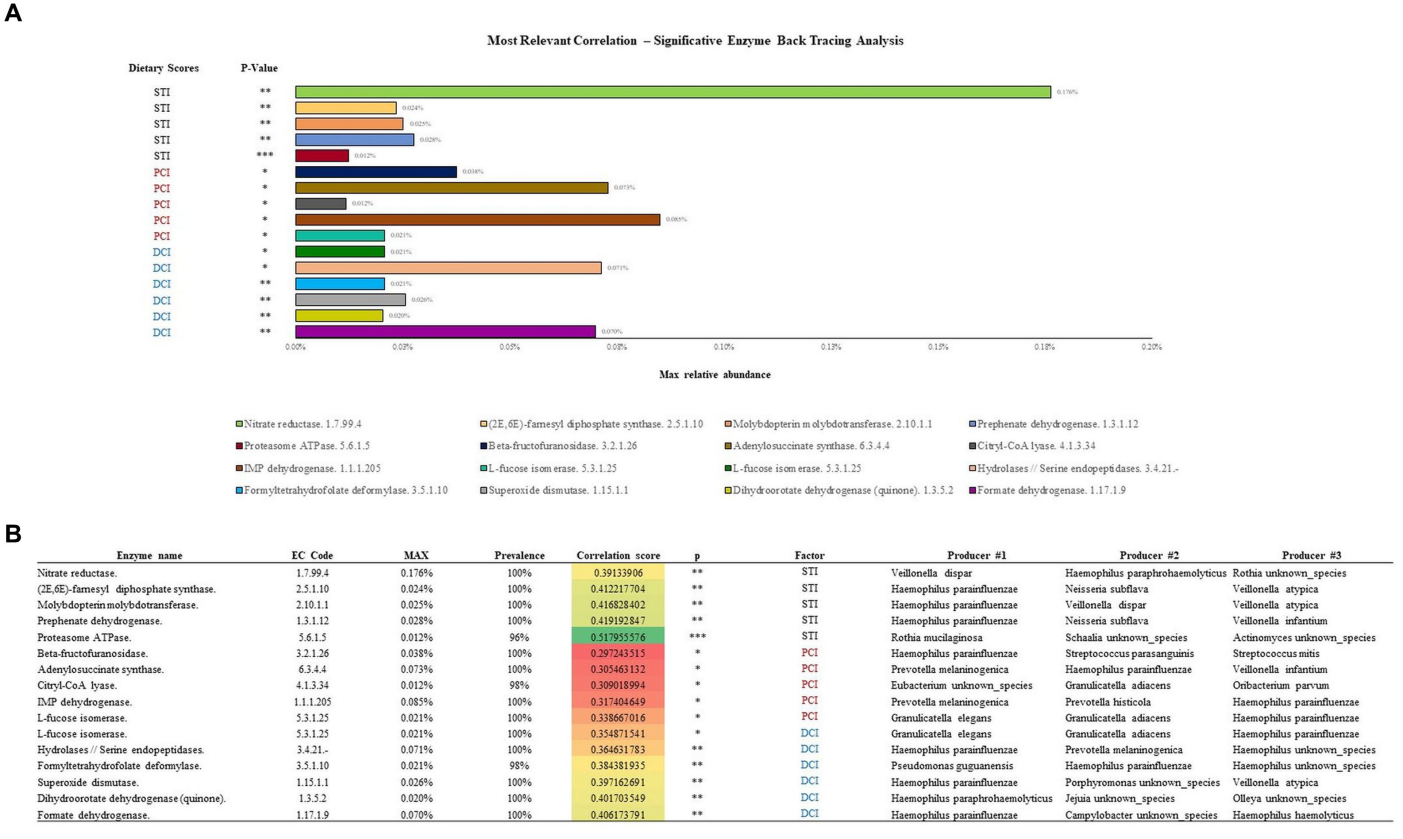
Enzymatic classes showing the highest correlations with STI, PCI and DCI are described in **(A)**. **(B)** Shows the bacterial species responsible for the enzymatic classes.

A subsequent back-tracing analysis was performed among the reactions encoded by these enzymes, showing the highest correlations with PCI, DCI and STI (five unique reactions for each; Figure 5). The enzyme L-fucose isomerase (EC 5.3.1.25) correlated positively with both DCI and PCI, and is hence listed twice in the figure. The back-tracing analysis showed that *Haemophilus* genus has a key role in defining the enzymes associated with PCI, DCI and STI, acting as the main taxa encoding for eight out of 16 of the enzymes analyzed (Figure 5B). Other main taxa encoding for some of these enzymes belong to *Pseudomonas*, *Veillonella*, *Rothia* and *Granulicatella* genera, followed by *Neisseria* and *Streptococcus* genera.

The enzymatic class correlating with STI with maximum relative abundance (0.176%) was Nitrate reductase (EC 1.7.99.4) encoded by *Veilonella dispar*. Similarly, IMP dehydrogenase (EC 1.1.1.205) correlated (0.085%) with PCI, encoded by *Prevotella melaninogenica*, the most common species in our samples, and Hydrolases/Serine endopeptidases (EC 3.4.21.-) was most abundantly correlated (0.071%) with DCI, encoded by *Haemophilus parainfluenzae*. Overall, *Haemophilus parainfluenzae* encoded the most enzymatic classes in relation to the dietary scores.

## 4 Discussion

Our findings based on saliva metagenome sequencing show a high inter-individual variability at the species level, which could be attributed to individual dietary habits, but less to central obesity in this age group. Central obesity is a clinically relevant marker of adiposity (Ross et al., 2020), yet, in our sample, bacterial composition differed only very modestly between groups with and without central obesity. In total, we observed 16 enzymatic classes that differed significantly between the groups. Contrary to this, habitual food consumption was highlighted as a potential modifier of bacterial enzymatic reactions in saliva. To our knowledge, no previous studies have addressed this topic.

### 4.1 Saliva microbiome and central obesity

*Pseudomonas* was the only genus that differed between the groups and was less abundant among children with central obesity compared to those without central obesity. The species-level analyses further verified that *Pseudomonas guguanensis* was less common and unclassified *Pseudomonas* fully absent in individuals with central obesity. In addition, the back-tracing analysis suggested that *P. guguanensis* contributes to formyltetrahydrofolate deformylase enzymatic class, which is a key enzyme in the pathway providing formate to the cell under aerobic growth conditions. This enzyme was also associated with dairy consumption in our sample, perhaps activating the same pathway. Indeed, slightly lower consumption of dairy products was marked among the group with central obesity compared with others, although this difference was not statistically significant.

*Prevotella* was the most common genus with prevalence of 29%, with similar abundances in the groups. However, certain species such as *P. oris*, *P. oulorum*, and *P. scopos* were fully absent in participants with central obesity. The most dominant species *Prevotella melaninogenica* and *P. histicola* were linked to the same enzymatic class IMP dehydrogenase which catalyzes the first committed, rate-limiting step in *de novo* guanine nucleotide biosynthesis in most organisms. Besides, *P. melaninogenica* also contributes to adenylosuccinate synthase, which catalyzes AMP’s *de novo* synthesis; both these enzymes were linked with the consumption of vegetables, fruits, and berries in our study. Previously, a high abundance of *Prevotella* was observed in the saliva of participants following plant-rich diets (Hansen et al., 2018; Daniele et al., 2021). In particular, the presence of *Prevotella* species correlated with high intake of dietary fiber (Daniele et al., 2021), which could be generally lower in individuals with central obesity (Parikh et al., 2012).

### 4.2 Saliva microbiome and habitual food consumption

The novelty of our study is that we demonstrated associations of habitual food consumption with the bacterial enzymatic profiles in saliva. We measured habitual diet using three dietary indices indicating weekly consumption frequencies of sweet treats, plants, and dairy.

Although dairy consumption associated with a very large enzymatic repertoire both positively (*n* = 43) and negatively (*n* = 88), the strongest modulator was the consumption of sweet treats, which had the highest correlation ratio among the three dietary scores as well as the most positive correlations. It also contributed to the most abundant nitrate reduction pathway through nitrate reductase. These findings resemble our recent results in another Fin-HIT sample employing 16S profiling on saliva microbiota; that study also highlighted the nitrate reduction pathway in frequent sugar consumers (Lommi et al., 2022). The pathway converts nitrate to nitrite, allowing further metabolism to nitric oxide (Rosier et al., 2020). Nitric oxide has physiological effects like vasodilation, and it contributes to innate immunity responses (Gantner et al., 2020), while in the mouth it increases the nitrate reduction capacity of the oral microbiota and is related with lower prevalence of caries (Doel et al., 2005). Specifically, nitrate reduction prevents acidification and cariogenic bacteria’s overgrowth by increasing lactate and ammonia production (Rosier et al., 2020). Based on the back-tracing analysis in our study, species responsible for nitrate reduction were *Veillonella dispar*, *Haemophilus paraprohaemolyticus* and unclassified *Rothia.*

Plant consumption was associated with the lowest number of enzymatic classes and showed a positive correlation with only seven enzymes, among them beta-fructofuranosidase and L-fucose isomerase, which are utilized to break down certain complex sugars from plant sources (Vanhooren and Vandamme, 1999; Kotwal and Shankar, 2009). This suggests that the enzymatic profiles of saliva microbiome adapt to the prevailing diet.

*Haemophilus parainfluenzae* was among the key producers of the enzymatic reactions related to these three dietary summary scores. Typically, *Haemophilus parainfluenzae* is considered pathogenic to humans and is related to infections in urban environment (Roslund et al., 2021). In our sample of children from densely populated areas of Finland, it was the second most common species in saliva microbiome.

### 4.3 Strengths and limitations

No difference in the beta diversity was observed between groups with and without central obesity, and only marginal differences in the abundance of some *Pseudomonas* and *Prevotella* species were noted. This could be due to small sample size and nonequal group sizes, but other explanations may exist as well. Initially, we selected samples with equal gender distribution with and without obesity based on BMI. However, since central obesity is considered a clinically more relevant measure of obesity than BMI, we divided the groups into with and without central obesity, leading to nonequal group sizes. Although we witnessed on average 20 cm larger waist circumference in the group with central obesity, the association with microbiome was superficial and likely explained by other factors instead. Here, we offer a plausible explanation with the habitual diet, but also other factors may contribute such as oral health. However, we have recently shown that the history of caries in this age group was not a determinant of saliva microbiome diversity nor composition (Manzoor et al., 2021) but associated positively with sugar-metabolizers such as *Leptorichia* and *Paludibacter* in sex-specific analyses. Nevertheless, our analysis was limited by the lack of information on active caries process.

Utilizing metagenomic sequencing is a strength of this study. It provides more detailed information than 16S profiling; classifying the taxa more accurately and allowing to reflect microbiome functions in terms of enzymatic classes and related pathways based on a detailed database on microbial species and their functions.

Our dietary summary scores rely on self-reported consumption of indicatory food items that do not provide a complete picture of the children’s diet. Furthermore, we had no exclusion criteria for participants; thus, some residual confounding may exist. Overall, the study should be considered a pilot study that provides observational data without full explanatory power.

## 5 Conclusion

Despite clinically relevant differences in waist circumference in participants, the saliva microbiota exhibited very modest differences in bacterial abundance and some in enzymatic classes according to central obesity. By contrast, habitual food consumption was associated with several enzymatic classes. Especially the role of sweet treat consumption was highlighted, as it contributed to nitrate reduction pathway through nitrate reductase produced by species *Veillonella dispar* and *Haemophilus parainfluenzae* among others. In adolescence, the contribution of lifestyle factors such as diet may have a bigger role on the saliva microbiome than central obesity *per se*.

## Data availability statement

The data presented in the study are deposited in the NCBI BioProject repository, accession number PRJNA1052614 (https://www.ncbi.nlm.nih.gov/bioproject/PRJNA1052614).

## Ethics statement

The studies involving humans were approved by the Coordinating Ethics Committee of the Hospital District of Helsinki and Uusimaa approved the study protocol (169/13/03/00/10). The studies were conducted in accordance with the local legislation and institutional requirements. Written informed consent for participation in this study was provided by the participant and his/her parent or legal guardian.

## Author contributions

NA: Conceptualization, Investigation, Writing – original draft, Writing – review & editing. FF: Formal analysis, Investigation, Visualization, Writing – original draft, Writing – review & editing. CT: Formal analysis, Writing – review & editing. SL: Investigation, Visualization, Writing – review & editing. MV: Formal analysis, Writing – review & editing. CM: Conceptualization, Writing – review & editing, Formal analysis, Investigation, Supervision. HV: Conceptualization, Formal analysis, Funding acquisition, Supervision, Writing – original draft, Writing – review & editing, Investigation, Visualization.

## Footnotes

^1^http://www.rstudio.com/

## References

[ref1] Bastian M. Heymann S. Jacomy M. (2009). Gephi: an open source software for exploring and manipulating networks. Proceedings of the International AAAI Conference on Web and Social Media, San Jose, CA, United States.

[ref2] Caspi R. Altman T. Billington R. Dreher K. Foerster H. Fulcher C. A.. (2014). The Meta Cyc database of metabolic pathways and enzymes and the BioCyc collection of pathway/genome databases. Nucleic Acids Res. 42, D459–D471. doi: 10.1093/NAR/GKT1103, PMID: 24225315 PMC3964957

[ref3] Chrzanowska M. Suder A. (2010). Changes in central fatness and abdominal obesity in children and adolescents from Cracow, Poland 1983-2000. Ann. Hum. Biol. 37, 243–253. doi: 10.3109/03014460903193237 19919496

[ref4] Coker M. O. Lebeaux R. M. Hoen A. G. Moroishi Y. Gilbert-Diamond D. Dade E. F.. (2022). Metagenomic analysis reveals associations between salivary microbiota and body composition in early childhood. Sci. Rep. 12:13075. doi: 10.1038/S41598-022-14668-Y, PMID: 35906254 PMC9338228

[ref5] Daniele S. Scarfò G. Ceccarelli L. Fusi J. Zappelli E. Biagini D.. (2021). The Mediterranean diet positively affects resting metabolic rate and salivary microbiota in human subjects: a comparison with the vegan regimen. Biology 10:1292. doi: 10.3390/biology10121292, PMID: 34943207 PMC8699008

[ref6] Deo P. N. Deshmukh R. (2019). Oral microbiome: unveiling the fundamentals. J. Oral Maxillofacial Pathol. 23, 122–128. doi: 10.4103/JOMFP.JOMFP_304_18, PMID: 31110428 PMC6503789

[ref7] Doel J. J. Benjamin N. Hector M. P. Rogers M. Allaker R. P. (2005). Evaluation of bacterial nitrate reduction in the human oral cavity. Eur. J. Oral Sci. 113, 14–19. doi: 10.1111/J.1600-0722.2004.00184.X, PMID: 15693824

[ref8] Figueiredo R. A. D. O. Simola-Ström S. Rounge T. B. Viljakainen H. Eriksson J. G. Roos E.. (2019). Cohort profile: the Finnish health in teens (fin-HIT) study: a population-based study. Int. J. Epidemiol. 48, 23–24H. doi: 10.1093/ije/dyy189, PMID: 30212855 PMC6380305

[ref9] Gantner B. N. LaFond K. M. Bonini M. G. (2020). Nitric oxide in cellular adaptation and disease. Redox Biol. 34:101550. doi: 10.1016/j.redox.2020.101550, PMID: 32438317 PMC7235643

[ref10] Garnett S. P. Baur L. A. Cowell C. T. (2011). The prevalence of increased central adiposity in Australian school children 1985 to 2007. Obesity Rev. 12, 887–896. doi: 10.1111/J.1467-789X.2011.00899.X, PMID: 21722299

[ref11] Hansen T. H. Kern T. Bak E. G. Kashani A. Allin K. H. Nielsen T.. (2018). Impact of a vegan diet on the human salivary microbiota. Sci. Rep. 8:5847. doi: 10.1038/s41598-018-24207-3, PMID: 29643500 PMC5895596

[ref12] Jacomy M. Venturini T. Heymann S. Bastian M. (2014). ForceAtlas2, a continuous graph layout algorithm for Handy network visualization designed for the Gephi software. PLoS One 9:e98679. doi: 10.1371/JOURNAL.PONE.0098679, PMID: 24914678 PMC4051631

[ref13] Kotwal S. M. Shankar V. (2009). Immobilized invertase. Biotechnol. Adv. 27, 311–322. doi: 10.1016/j.biotechadv.2009.01.009 19472508

[ref14] Lommi S. Figueiredo R. A. D. O. Tuorila H. Viljakainen H. (2020). Frequent use of selected sugary products associates with thinness, but not overweight during preadolescence: a cross-sectional study. Br. J. Nutr. 124, 631–640. doi: 10.1017/S0007114520001361, PMID: 32312332 PMC7525105

[ref15] Lommi S. Manzoor M. Engberg E. Agrawal N. Lakka T. A. Leinonen J.. (2022). The composition and functional capacities of saliva microbiota differ between children with low and high sweet treat consumption. Front. Nutr. 9:543. doi: 10.3389/FNUT.2022.864687/BIBTEX PMC9085455 35558746

[ref16] Lu Y. Yuan X. Wang M. He Z. Li H. Wang J.. (2022). Gut microbiota influence immunotherapy responses: mechanisms and therapeutic strategies. J. Hematol. Oncol. 15:47. doi: 10.1186/S13045-022-01273-9, PMID: 35488243 PMC9052532

[ref17] Manzoor M. Lommi S. Furuholm J. Sarkkola C. Engberg E. Raju S.. (2021). High abundance of sugar metabolisers in saliva of children with caries. Sci. Rep. 11, 4424–4410. doi: 10.1038/s41598-021-83846-1, PMID: 33627735 PMC7904847

[ref18] McCarthy H. D. Jarrett K. V. Emmett P. M. Rogers I. (2005). Trends in waist circumferences in young British children: a comparative study. Int. J. Obesity 29, 157–162. doi: 10.1038/SJ.IJO.0802849, PMID: 15570313

[ref19] Milani C. Lugli G. A. Fontana F. Mancabelli L. Alessandri G. Longhi G.. (2021). METAnnotatorX2: a comprehensive tool for deep and shallow metagenomic data set analyses. mSystems 6:e0058321. doi: 10.1128/MSYSTEMS.00583-21, PMID: 34184911 PMC8269244

[ref20] Mokha J. S. Srinivasan S. R. DasMahapatra P. Fernandez C. Chen W. Xu J.. (2010). Utility of waist-to-height ratio in assessing the status of central obesity and related cardiometabolic risk profile among normal weight and overweight/obese children: the Bogalusa heart study. BMC Pediatr. 10:73. doi: 10.1186/1471-2431-10-73 20937123 PMC2964659

[ref21] Nishida A. Inoue R. Inatomi O. Bamba S. Naito Y. Andoh A. (2018). Gut microbiota in the pathogenesis of inflammatory bowel disease. Clin. J. Gastroenterol. 11, 1–10. doi: 10.1007/S12328-017-0813-5 29285689

[ref22] Parikh S. Pollock N. K. Bhagatwala J. Guo D.-H. Gutin B. Zhu H.. (2012). Adolescent fiber consumption is associated with visceral fat and inflammatory markers. J. Clin. Endocrinol. Metab. 97, E1451–E1457. doi: 10.1210/jc.2012-1784, PMID: 22593589 PMC3410273

[ref23] Räisänen L. Lommi S. Engberg E. Kolho K. L. Viljakainen H. (2022). Central obesity in school-aged children increases the likelihood of developing paediatric autoimmune diseases. Pediatric Obesity 17:e12857. doi: 10.1111/IJPO.12857 34608761 PMC9285017

[ref24] Raju S. C. Lagström S. Ellonen P. de Vos W. M. Eriksson J. G. Weiderpass E.. (2018). Reproducibility and repeatability of six high-throughput 16S rDNA sequencing protocols for microbiota profiling. J. Microbiol. Methods 147, 76–86. doi: 10.1016/j.mimet.2018.03.003 29563060

[ref25] Raju S. C. Lagström S. Ellonen P. de Vos W. M. Eriksson J. G. Weiderpass E.. (2019). Gender-specific associations between saliva microbiota and body size. Front. Microbiol. 10:767. doi: 10.3389/fmicb.2019.00767, PMID: 31024514 PMC6467948

[ref26] Rosier B. T. Moya-Gonzalvez E. M. Corell-Escuin P. Mira A. (2020). Isolation and characterization of nitrate-reducing bacteria as potential probiotics for oral and systemic health. Front. Microbiol. 11:555465. doi: 10.3389/FMICB.2020.555465, PMID: 33042063 PMC7522554

[ref27] Roslund M. I. Puhakka R. Nurminen N. Oikarinen S. Siter N. Grönroos M.. (2021). Long-term biodiversity intervention shapes health-associated commensal microbiota among urban day-care children. Environ. Int. 157:106811. doi: 10.1016/J.ENVINT.2021.106811, PMID: 34403882

[ref28] Ross R. Neeland I. J. Yamashita S. Shai I. Seidell J. Magni P.. (2020). Waist circumference as a vital sign in clinical practice: a consensus statement from the IAS and ICCR working group on visceral obesity. Nat. Rev. Endocrinol. 16, 177–189. doi: 10.1038/S41574-019-0310-7, PMID: 32020062 PMC7027970

[ref29] Sarkkola C. Rounge T. B. Simola-Ström S. Von Kraemer S. Roos E. Weiderpass E. (2016). Validity of home-measured height, weight and waist circumference among adolescents. Eur. J. Pub. Health 26, 975–977. doi: 10.1093/EURPUB/CKW133, PMID: 27578829

[ref30] Vanhooren P. Vandamme E. (1999). L-Fucose: occurrence, physiological role, chemical, enzymatic and microbial synthesis. J. Chem. Technol. Biotechnol. 74, 479–497. doi: 10.1002/(SICI)1097-4660(199906)74:6<479::AID-JCTB76>3.0.CO;2-E

[ref31] Vereecken C. A. Maes L. (2003). A Belgian study on the reliability and relative validity of the health behaviour in school-aged children food-frequency questionnaire. Public Health Nutr. 6, 581–588. doi: 10.1079/PHN2003466 14690039

[ref32] Vereecken C. A. Rossi S. Giacchi M. V. Maes L. (2008). Comparison of a short food-frequency questionnaire and derived indices with a seven-day diet record in Belgian and Italian children. Int. J. Public Health 53, 297–305. doi: 10.1007/S00038-008-7101-6 19112592

[ref33] Wade W. G. (2021). Resilience of the oral microbiome. Periodontol. 86, 113–122. doi: 10.1111/PRD.12365 33690989

[ref34] World Health Organization. (2022). WHO European regional obesity report 2022. Copenhagen: WHO Regional Office for Europe.

