# Supplementary material for: Associations of central obesity and habitual food consumption with saliva microbiota and its enzymatic profiles – a pilot study in Finnish children
Source: Front Microbiol. 2024 Jan 8;14:1323346. doi: 10.3389/fmicb.2023.1323346 (PMC10801001; doi:10.3389/fmicb.2023.1323346)
Supplement: Supplementary file 1 [file Data_Sheet_1.DOCX]

Supplementary Material

# Supplementary Tables

## Table 1

| **Supplementary Table 1.** Sequenced reads summary report. | | |  |  |
| --- | --- | --- | --- | --- |
|  |  | |  |  |
| **Sample** | **Sequenced reads** | **High quality reads** | | **Retained reads after Homo sapiens filtering** |
| **TSAZBAIRYLWL** | 1825260 | 1817446 | | 114167 |
| **TSBKNBKBPWMJ** | 2072698 | 2060888 | | 138000 |
| **TSBMNQBQSPMS** | 2150898 | 2138881 | | 239107 |
| **TSCRGRJTPNSP** | 1829411 | 1819040 | | 120995 |
| **TSCYFZZZZRYH** | 1354967 | 1347598 | | 98507 |
| **TSDKBTTXEBBF** | 1864885 | 1855116 | | 285222 |
| **TSDQZCRWWFET** | 1488772 | 1481079 | | 109490 |
| **TSDTZLNDQIZQ** | 1604196 | 1597454 | | 96591 |
| **TSEDGQVBUUTW** | 2105193 | 2093961 | | 129384 |
| **TSEJUFAJXEIT** | 2692930 | 2682668 | | 956252 |
| **TSFYDQIYNHUE** | 1802319 | 1794030 | | 124705 |
| **TSGBSGBIMEHV** | 2075434 | 2064947 | | 423216 |
| **TSGCQSBGREID** | 2410739 | 2399571 | | 173562 |
| **TSGLLKAJIFBG** | 2132504 | 2120979 | | 111429 |
| **TSHGADDQSMDA** | 2288791 | 2276732 | | 198723 |
| **TSHHFJUPJQSC** | 1918803 | 1910941 | | 326445 |
| **TSHPZSPARFUP** | 1330699 | 1323793 | | 91414 |
| **TSHWQGSUERYT** | 1789915 | 1782721 | | 602674 |
| **TSIAADMPHZFD** | 1988983 | 1978976 | | 130439 |
| **TSIBBBRFZXPV** | 2028372 | 2018917 | | 378301 |
| **TSLVALUMBFDF** | 1719912 | 1710935 | | 91976 |
| **TSMAENPDTIPG** | 2667056 | 2657225 | | 1200362 |
| **TSMCEJAZXXEJ** | 1989380 | 1980675 | | 655644 |
| **TSMCKPMFKRYF** | 2462460 | 2449482 | | 105710 |
| **TSMNTLLXYMIA** | 2583684 | 2569873 | | 298531 |
| **TSMUJWHIPMFQ** | 2031101 | 2019531 | | 168752 |
| **TSMURHSULPGN** | 1752108 | 1745325 | | 607987 |
| **TSNFAIVWXBDP** | 1489621 | 1486839 | | 1427437 |
| **TSNIBKJHXNKY** | 2031688 | 2021546 | | 249427 |
| **TSNKGTVSTTTS** | 1901414 | 1892547 | | 404155 |
| **TSNXVGFTFDNZ** | 1619539 | 1612888 | | 346766 |
| **TSQXJJYSJBEW** | 1539080 | 1531666 | | 145689 |
| **TSRINVAFZHEH** | 1927313 | 1915951 | | 46690 |
| **TSRVLBZQNJLR** | 2238134 | 2226239 | | 306217 |
| **TSSHTSLNSUHP** | 1343483 | 1335722 | | 33640 |
| **TSTIKWEKFICD** | 2152134 | 2141825 | | 438716 |
| **TSTYNAVHKWRK** | 1796393 | 1786953 | | 81523 |
| **TSUFXTRUEDUG** | 1909587 | 1900745 | | 169559 |
| **TSULBSBNMKNS** | 2071172 | 2063021 | | 758777 |
| **TSVGRSWQZFSY** | 1915160 | 1905012 | | 89916 |
| **TSWBUZHGABVZ** | 1970109 | 1961542 | | 413514 |
| **TSWKTGLZWTJB** | 1560212 | 1551497 | | 75901 |
| **TSWQBQFZIALI** | 2222208 | 2210127 | | 118430 |
| **TSXJUTEBKTSU** | 1879163 | 1869788 | | 169306 |
| **TSYFDVNMISRZ** | 2047609 | 2038225 | | 422686 |
| **TSYITNUGQEXQ** | 2122366 | 2110507 | | 111119 |
| **TSYKBACMLXRV** | 2420762 | 2407232 | | 85704 |
| **TSZBVNQNYHNP** | 2297708 | 2284676 | | 95981 |
| **TSZEEWRNUJZM** | 2419457 | 2406640 | | 215627 |
| **TSZLNPEVTXVG** | 1813162 | 1803520 | | 94031 |

## Table 2

| **Supplementary Table 2**. The frequency of the top 20 most common species in saliva 50 adolescents | | | | |
| --- | --- | --- | --- | --- |
|  | Mean % | SD % | Minimum % | Maximum % |
| *Prevotella melaninogenica* | 11.72 | 8.05 | 1.42 | 38.72 |
| *Haemophilus parainfluenzae* | 8.03 | 4.77 | 0.00 | 21.97 |
| *Prevotella histicola* | 5.54 | 8.14 | 0.00 | 49.17 |
| *Porphyromonas pasteri* | 4.95 | 4.70 | 0.00 | 19.58 |
| *Veillonella nakazawae* | 4.29 | 2.73 | 0.00 | 11.60 |
| *Veillonella atypica* | 3.54 | 2.95 | 0.00 | 12.84 |
| *Prevotella unknown_species* | 2.92 | 1.42 | 0.67 | 8.02 |
| *Haemophilus unknown_species* | 2.75 | 1.68 | 0.00 | 7.17 |
| *Neisseria sicca* | 2.67 | 2.73 | 0.00 | 11.98 |
| *Streptococcus mitis* | 2.51 | 2.25 | 0.00 | 8.39 |
| *Morococcus cerebrosus* | 2.41 | 2.22 | 0.00 | 7.61 |
| *Haemophilus haemolyticus* | 2.40 | 1.80 | 0.00 | 8.28 |
| *Prevotella jejuni* | 2.36 | 3.08 | 0.00 | 14.60 |
| *Streptococcus unknown_species* | 2.32 | 1.48 | 0.00 | 7.19 |
| *Porphyromonas unknown_species* | 1.95 | 1.64 | 0.00 | 5.89 |
| *Neisseria subflava* | 1.91 | 2.29 | 0.00 | 8.69 |
| *Veillonella rogosae* | 1.90 | 1.59 | 0.00 | 5.67 |
| *Rothia mucilaginosa* | 1.89 | 2.70 | 0.00 | 14.49 |
| *Campylobacter unknown_species* | 1.75 | 1.34 | 0.00 | 6.19 |
| *Prevotella pallens* | 1.72 | 1.56 | 0.00 | 5.68 |


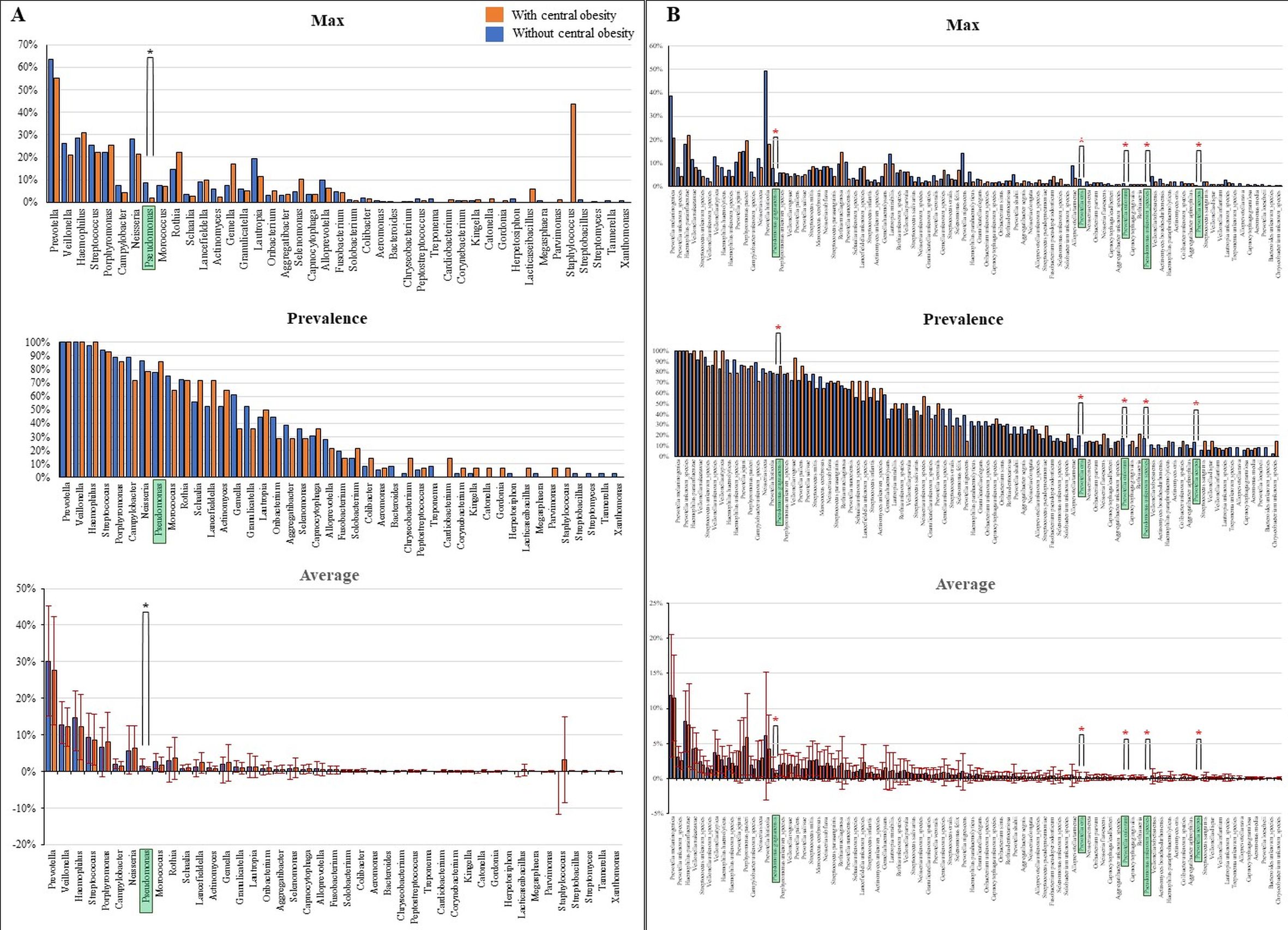


**Supplementary Figure 1:** The maximum presence in a sample (Max), total presence among all samples (Prevalence), and Average presence in the whole sample (Average) is shown here for **(A)** genus and **(B)** species composition between the groups with and without central obesity. The genera and species with significant differences between the groups are marked with green boxes.
